# Complete genome sequences of two strains of *Treponema pallidum* subsp. *pertenue* from Ghana, Africa: Identical genome sequences in samples isolated more than 7 years apart

**DOI:** 10.1371/journal.pntd.0005894

**Published:** 2017-09-08

**Authors:** Michal Strouhal, Lenka Mikalová, Pavla Havlíčková, Paolo Tenti, Darina Čejková, Ivan Rychlík, Sylvia Bruisten, David Šmajs

**Affiliations:** 1 Department of Biology, Faculty of Medicine, Masaryk University, Kamenice 5, Brno, Czech Republic; 2 Department of Immunology, Veterinary Research Institute, Hudcova 296/70, Brno, Czech Republic; 3 Regional Laboratory for Public Health, Nieuwe Achtergracht 100, Amsterdam, The Netherlands; University of Washington, UNITED STATES

## Abstract

**Background:**

*Treponema pallidum* subsp. *pertenue* (TPE) is the causative agent of yaws, a multi-stage disease, endemic in tropical regions of Africa, Asia, Oceania, and South America. To date, four TPE strains have been completely sequenced including three TPE strains of human origin (Samoa D, CDC-2, and Gauthier) and one TPE strain (Fribourg-Blanc) isolated from a baboon. All TPE strains are highly similar to *T*. *pallidum* subsp. *pallidum* (TPA) strains. The mutation rate in syphilis and related treponemes has not been experimentally determined yet.

**Methodology/Principal findings:**

Complete genomes of two TPE strains, CDC 2575 and Ghana-051, that infected patients in Ghana and were isolated in 1980 and 1988, respectively, were sequenced and analyzed. Both strains had identical consensus genome nucleotide sequences raising the question whether TPE CDC 2575 and Ghana-051 represent two different strains. Several lines of evidence support the fact that both strains represent independent samples including regions showing intrastrain heterogeneity (13 and 5 intrastrain heterogeneous sites in TPE Ghana-051 and TPE CDC 2575, respectively). Four of these heterogeneous sites were found in both genomes but the frequency of alternative alleles differed. The identical consensus genome sequences were used to estimate the upper limit of the yaws treponeme evolution rate, which was 4.1 x 10^−10^ nucleotide changes per site per generation.

**Conclusions/Significance:**

The estimated upper limit for the mutation rate of TPE was slightly lower than the mutation rate of *E*. *coli*, which was determined during a long-term experiment. Given the known diversity between TPA and TPE genomes and the assumption that both TPA and TPE have a similar mutation rate, the most recent common ancestor of syphilis and yaws treponemes appears to be more than ten thousand years old and likely even older.

## Author summary

The causative agent of yaws, *Treponema pallidum* subsp. *pertenue* (TPE), belongs to a group of uncultivable treponemes causing several human and animal infections. Yaws is a multi-stage disease which is endemic in tropical regions of Africa, Asia, Oceania, and South America. In this study, whole genome sequences of two TPE strains (CDC 2575 and Ghana-051) isolated from patients infected in Ghana, Africa, were determined. Despite being isolated more than 7 years apart (1980 and 1988), both TPE strains had an identical consensus whole genome nucleotide sequence, although showing differences in 14 intrastrain heterogeneous sites. To support the independent character of both strains, re-analysis of available lab records, clinical data and sequencing of the contaminating rabbit chromosomal DNA was performed and revealed differences in TPE Ghana-051 and CDC 2575 samples. The identical genome sequence and the time between isolation of both TPE strains was used to estimate the upper limit of the yaws treponeme evolution rate, which was 4.1 x 10^−10^ per site per generation, a mutation rate that is slightly lower than the experimentally determined *E*. *coli* mutation rate. Given the obtained data, the most recent common ancestor of syphilis and yaws treponemes is likely to be more than ten thousand years old. However, since our estimation is an upper limit of the evolution rate, one can assume that the real evolutionary rate could have been even slower, and the time to the most recent common ancestor of syphilis and yaws treponemes even longer.

## Introduction

*Treponema pallidum* subsp. *pertenue* (TPE) is the causative agent of yaws, a multi-stage disease transmitted through direct skin contact between children or young adults; it is characterized by skin nodules and ulcerations and later accompanied by joint, soft tissue, and bone affections (reviewed in [1]).

To date, four TPE strains have been completely sequenced including three TPE strains of human origin (Samoa D, CDC-2, and Gauthier) [2] and one TPE strain (Fribourg-Blanc) isolated from a baboon (*Papio papio*) in West Africa [3]. Compared to syphilis-causing strains of *T*. *pallidum* subsp. *pallidum* (TPA), the observed genetic differences between TPA and TPE represent less than 0.2% of the total genome sequence length [2]. In addition, TPE strains have been shown to be closely related to the *T*. *pallidum* subsp. *endemicum* (TEN) strain Bosnia A [4].

The origin of syphilis and other treponematoses (i.e., diseases caused by TPE and TEN) remain enigmatic and historically there have been several hypotheses about the origin of these diseases (reviewed in [5]). One of the limitations was the fact that the mutation rate in syphilis and related treponemes was unknown, although estimates have been published based on either paleopathological findings [6] or on phylogenetic analyses of TPA strains/isolates with known isolation dates [7].

In this communication, we compared the complete genome sequences of two strains of TPE which came from infected patients living in Ghana in 1980 and 1988. While there were more than 7 years between isolation dates of the two treponemal strains, the assembled consensus genome sequences of both TPE strains were identical, although both strains exhibited intrastrain heterogeneity at a limited number of nucleotide positions. These data were used to estimate the upper limit of the yaws treponeme evolution rate, as well as evaluate the possible evolutionary implications.

## Methods

### Ethics statement

No vertebrate animals were used in the study. The study was approved by the ethics committee of the Faculty of Medicine, Masaryk University. The research was conducted in accordance with the Czech law standards.

### Strains used in this study

Two TPE strains (TPE Ghana-051 and CDC 2575) were used in this study. In 1980, TPE strain CDC 2575 was isolated in Akorabo, Ghana, from a 6-year-old female patient from a papillomatous lesion and inoculated into hamsters [8], and later propagated in New Zealand White rabbits [9]. TPE strain Ghana-051 was isolated in 1988 from a papillomatous lesion of 9-year-old girl who was infected at an unspecified location in Ghana six months prior to admission to Sophia Children's Hospital in Rotterdam, The Netherlands [10]. An extract from a biopsy of this patient in secondary yaws stage was inoculated into New Zealand White rabbits in the Netherlands [10]. DNA from the TPE strains CDC 2575 was isolated from rabbit testes lysates containing 10^7^ treponemes per ml. TPE Ghana-051 was provided as a DNA isolate that was previously isolated directly from rabbit testes containing 10^9^ treponemes per ml. Both strains came from the laboratory strain collection of Dr. Gerda Noordhoek.

### Amplification of TPE genomic DNA

The DNA of both TPE strains was isolated from treponemes obtained during experimental infection of New Zealand White rabbits. The genomic DNA was amplified using the multiple displacement amplification approach (REPLI-g kit, QIAGEN, Valencia, CA, USA) according to the manufacturer’s instructions and then used as a template (50x diluted) for pooled segment genome sequencing (PSGS) as described previously [2–4, 11]. Briefly, the TPE DNA was amplified with PrimeSTAR GXL DNA Polymerase (Takara Bio Inc., Otsu, Japan) with 278 pairs of specific primers to obtain overlapping PCR products (S1 Table) covering the entire genome in both isolates. PCR products were amplified using touchdown PCR at the following cycling conditions: initial denaturation at 94°C for 1 min; 8 cycles: 98°C for 10 s, 68°C for 15 s (annealing temperature gradually reduced by 1°C/every cycle), and 68°C for 6 min; 35 cycles: 98°C for 10 s, 61°C for 15 s, and 68°C for 6 min (43 cycles in total); followed by the final extension at 68°C for 7 min. PCR products were subsequently purified using a QIAquick PCR Purification Kit (QIAGEN, Valencia, CA, USA) and mixed in equimolar amounts into four distinct pools (S1 Table). Prior to Illumina sequencing, the PCR products from each pool were labeled with multiplex identifier (MID) adapters and sequenced as four different samples.

### DNA sequencing and *de novo* assembly of the TPE genomes

Illumina Nextera XT library preparation and sequencing on a NextSeq 500 (2x150 bp) was performed at CEITEC (Brno, Czech Republic). The results were: (1) for CDC 2575, 15,629,124 paired reads, 4,688,737,200 total bases, with an average coverage depth of 1028x, and (2) for Ghana-051, 13,194,731 paired reads, 3,958,419,300 total bases, with an average coverage depth of 868x. The sequencing results are summarized in S2 Table. Resultant data were pre-processed with Trimmomatic (0.32) [12]. Low quality bases were removed with a sliding window having a length of 4, with an average quality of at least Phred = 17. After pre-processing, sequencing reads shorter than 50 bp were removed.

The Illumina sequencing reads were handled separately with respect to the 4 distinct pools and were separately assembled *de novo* using SeqMan NGen v4.1.0 software (DNASTAR, Madison, WI, USA). A total of 470, 783, 607, and 667 contigs for Ghana-051 and 72, 348, 57, and 215 contigs for TPE CDC 2575 were obtained for Pools 1–4, respectively. Alternatively, all Illumina sequencing reads were mapped to the TPE Samoa D genome (GenBank Acc. No. CP002374.1). All genome gaps and discrepancies were resolved by location-specific PCR products that were Sanger sequenced. Altogether, 26 and 23 regions of the Ghana-051 and CDC 2575 genome were Sanger sequenced, respectively. The individual overlapping pool sequences were joined together, resulting in the complete genome sequence of both isolates.

### Identification of genetic heterogeneity within TPE genomes

The individual Illumina reads were mapped to the final version of the corresponding complete genome sequences using SeqMan NGen v4.1.0 software (DNASTAR, Madison, WI, USA) with default parameters and requiring at least a 93% read identity relative to the reference genome. To determine the frequency of each nucleotide (allele frequency) in every single genome position, the haploid bayesian method was used for SNP calculation with default parameters using SeqMan NGen v4.1.0 software (DNASTAR, Madison, WI, USA). Nucleotide positions located within homopolymeric tracts (defined as a stretch of 6 or more identical nucleotides) were omitted from the analysis. Chromosomal loci showing genetic heterogeneity within TPE genomes were defined as those containing more than 8% alternative reads in regions having coverage greater than 100x. The resulting candidate sites for heterogeneous nucleotide positions were subsequently visually inspected using SeqMan NGen v4.1.0 software (DNASTAR, Madison, WI, USA).

### Gene identification, annotation, and classification

Geneious software v5.6.5 [13] was used for gene annotations, as described previously [4]. Genes were tagged with TPEGH051_ and TPECDC2575_ prefixes with the locus tag numbering corresponding to the tag numbering of the orthologous genes annotated in the TPE CDC-2 genome ([2]; GenBank Acc. No. CP002375.1). As in other TPE genomes, *tpr*K showed intrastrain variability [14] and the corresponding nucleotide positions were denoted as “N” in the complete genome sequences. For proteins with unpredicted functions, a gene size limit of 150 bp was applied.

### Analysis of whole genome sequences

In addition to the Ghana-051 and CDC 2575 genome sequences, whole genome nucleotide sequences of 4 TPE strains, i.e., Samoa D (CP002374.1), CDC-2 (CP002375.1), Gauthier (CP002376.1), Fribourg-Blanc (CP003902.1), and TEN Bosnia A strain (CP007548.1) were used for construction of a phylogenetic tree. Whole genome alignment was constructed using SeqMan software (DNASTAR, Madison, WI, USA) and the unrooted phylogenetic tree was constructed from the whole genome sequence alignment using the Maximum Likelihood method and MEGA7 software [15].

### Sequencing of rabbit DNA

Trimmed sequencing reads that passed quality threshold for both TPE Ghana-051 and CDC 2575 were mapped to the rabbit reference chromosome 1 (CM000790.1) using BWA-MEM (version 0.7.5a; [16]) software with default parameters. Altogether, sequencing of Ghana-051 and CDC 2575 resulted in 2,150,741 and 1,731,654 reads that aligned to the rabbit DNA, respectively. Single nucleotide variants in the rabbit chromosome-aligned sequences were detected using SAMtools (version 0.1.19; [17]) and VarScan (version v2.4.2; [18]) software by setting a minimum read depth of 8, strand filter on, and a mutant frequency of 100%. Altogether, 21 positions in the rabbit DNA were found to be heterogeneous between the Ghana-051 and CDC 2575 samples.

A region differing in 7 nucleotide positions and spanning approximately 1 kb was selected and analyzed in additional TPE samples containing rabbit DNA with one pair of primers including 5’-AAAGCCCCTTTGCCTAGTCC-3’ (positions 53848016–53848035 in the genome of *Oryctolagus cuniculus* (rabbit) according to reference sequence CM000790.1) and 5’-CCTGCGGCCCCATTATTGTA-3’ (positions 53849015–53848996 in CM000790.1). The reaction mixture contained 2.5 μl of ThermoPol Reaction Buffer, 0.5 μl of 10 mM deoxynucleoside triphosphate (dNTP) mixture, 0.25 μl of each primer (100 pmol/μl), 2 μl of the DNA-containing sample and 0.1 μl of *Taq* DNA polymerase (5,000 U/ml; New England BioLabs, Ipswich, MA, USA). The reaction mixture was supplemented with PCR-grade water to a final volume of 25 μl. The DNA was amplified under the following cycling conditions: denaturation at 94°C for 2 min; 35 cycles: 94°C for 15 s, 57.5°C for 15 s, 68°C for 1.25 min; followed by the final extension at 68°C for 2 min. The PCR products were purified using a QIAquick PCR Purification Kit (QIAGEN, Valencia, CA, USA) and sequenced using the Sanger method (GATC Biotech, Germany).

### Nucleotide sequence accession numbers

The complete genome sequences of the TPE Ghana-051 and CDC 2575 strains were deposited in the GenBank under accession number CP020365 and CP020366, respectively. The sequenced chromosomal rabbit DNA from TPE Ghana-051, CDC 2575, CDC-2, and Sei Geringging samples were deposited in GenBank under accession numbers KY972336, KY972337, KY972338, and KY972339, respectively.

## Results

### Isolation of TPE strains

**Fig 1:**
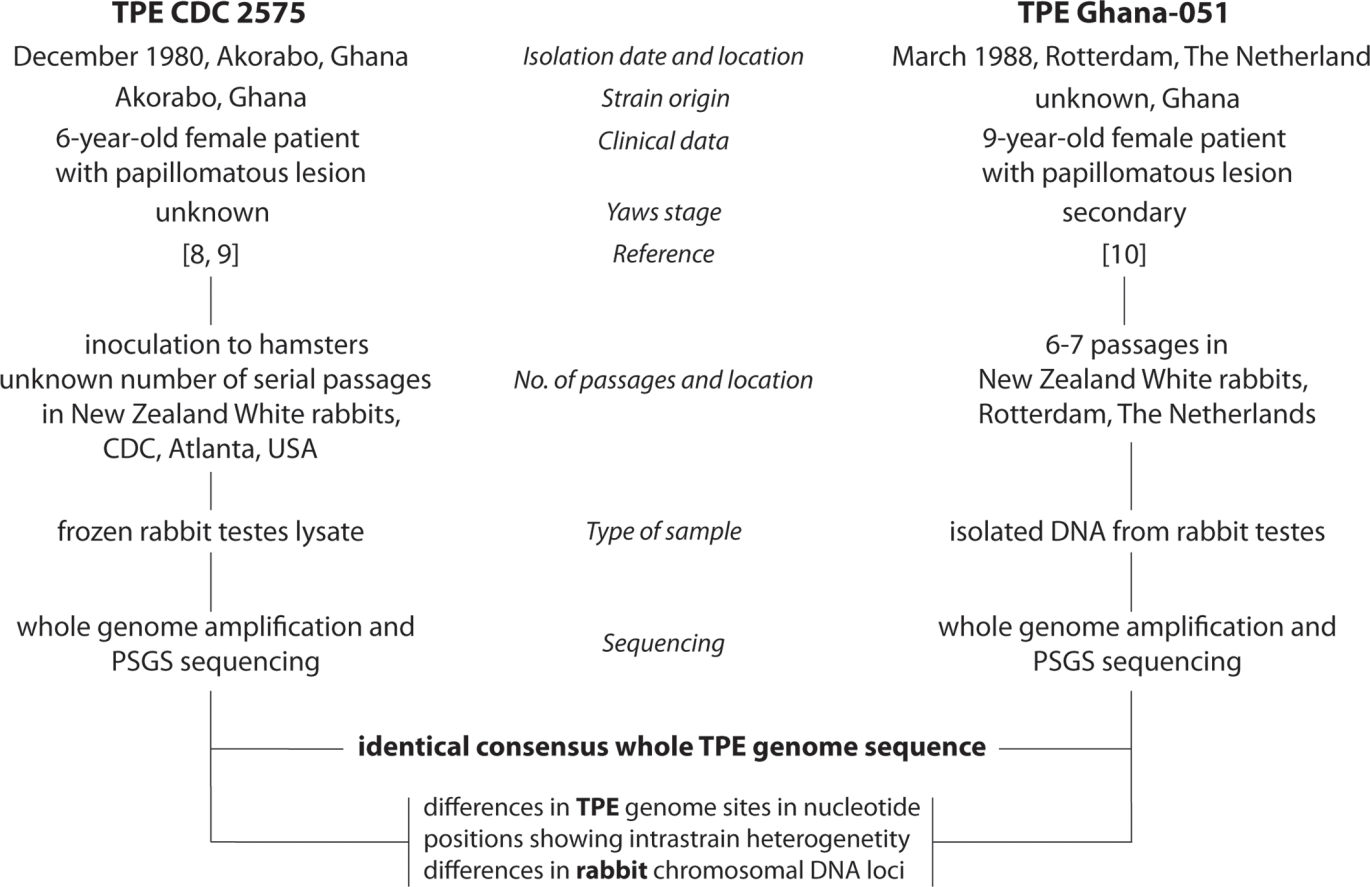
An origin of the analyzed samples including the time and place of isolation, clinical data and the number of passages in experimental animals. Both patients with TPE infection were infected in Ghana and the time between sample collections was 7 years and 3 months.

Both TPE strains (CDC 2575 and Ghana-051) used in this study were isolated from papillomatous lesions of young patients of comparable age (6 and 9 years old, respectively), and both patients were infected in Ghana, West Africa. While TPE strain CDC 2575 was isolated in Akorabo, Ghana, in 1980 [8, 9], TPE strain Ghana-051 was isolated in 1988 after its introduction, via an infected patient, to the Netherlands [10]. The time and place of the isolations, clinical data, as well as the species of experimentally infected animals and the known number of passages, are shown in Fig 1.

While Liska et al. [8] is provided as an original reference for TPE CDC 2575, this strain is not specifically mentioned in this study. According to the lab record from Dr. Gerda Noordhoek, CDC 2575 was provided by Dr. Peter Perine from CDC, one of the coauthors of Liska et al. [8] study, and strain details referred to Liska et al. [8]. Moreover, clinical data from Dr. Noordhoek´s notebook are in accordance with the description of the patient no. 1 in the Liska et al. [8] study (see Table 1 in Liska et al. [8]). Therefore, CDC 2575 represents one of the three successfully hamster propagated samples in the Liska et al. [8] study. The other two TPE strains, named as CDC-1 and CDC-2, were successfully transferred to rabbits and the third isolate (likely CDC 2575) was lost [8]. It is not clear if CDC 2575 represents the lost and perhaps again found sample. Nevertheless, the mislabeling or swapping of CDC 2575 with CDC-2 or CDC-1 can be excluded since all these strains differ in the genomic sequence [2].

In the original article by Engelkens et al. [10], strain Ghana-051 is also not specifically mentioned. However, according to the lab record of Dr. Noordhoek containing clinical data and reference, TPE Ghana-051 is the same strain as described by Engelkens et al. [10].

### Whole genome sequencing of TPE Ghana-051 and TPE CDC 2575

**Fig 2:**
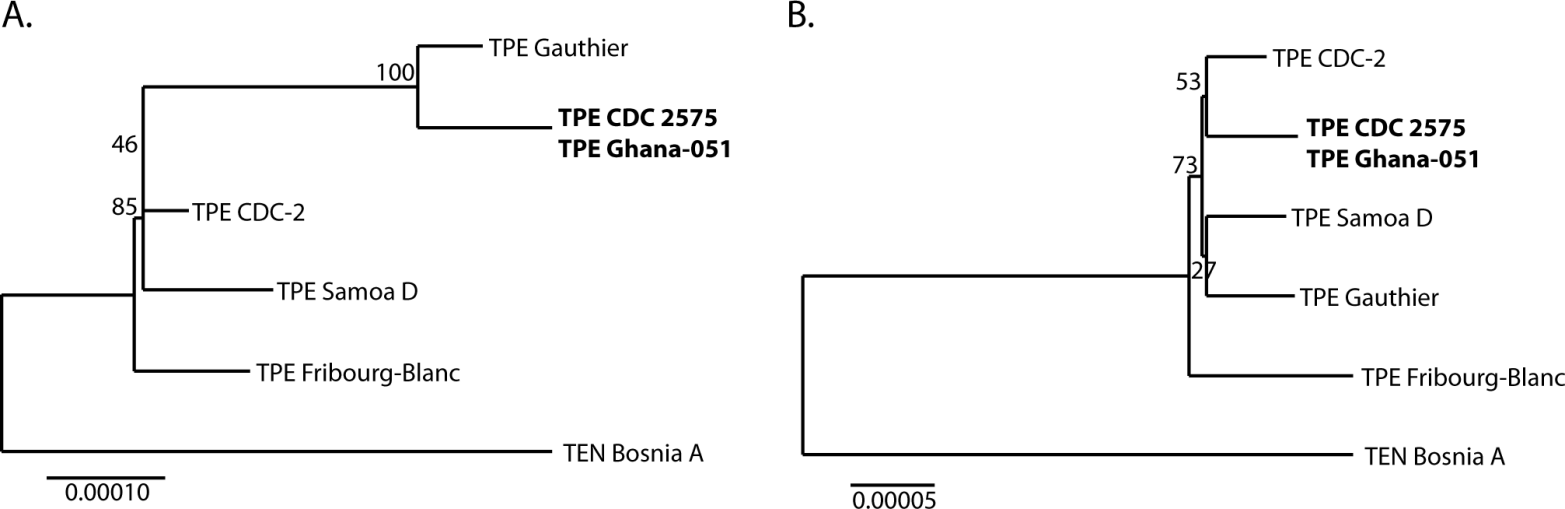
An unrooted tree constructed from whole genome sequence alignments from available TPE and TEN genomes. The tree was constructed using the Maximum Likelihood method based on Tamura-Nei model and MEGA7 software [15]. Bootstrap values based on 1,000 replications are shown next to the branches. **A.** An unrooted tree constructed from the whole genome sequence alignment of TPE and TEN genome sequences. The bar scale corresponds to a difference of 0.00010 nucleotides. **B.** An unrooted tree constructed from the whole genome sequence alignment of TPE and TEN genome sequences. The bar scale corresponds to a difference of 0.00005 nucleotides. *tprK* sequences, both rRNA operons, *tprD*, *arp*, and TP0470 genes were omitted from the analysis. Deletion of these regions resulted in a modified tree topology due to: (1) differences between the two possible constitutional states for the rRNA operons [19], (2) the presence of two *tprD* alleles in the TPE population (*tprD* and *tprD2*; [20]), and (3) differences in the number of repetitions in the *arp* and TP0470 genes.

Both TPE Ghana-051 and TPE CDC 2575 genomes were sequenced using the previously described PSGS approach, originally described by Weinstock et al. [11] and later modified by others [2–4]. The average coverage of Illumina reads reached 868x and 1028x for TPE Ghana-051 and TPE CDC 2575 genome, respectively. The two sequencing projects were performed separately and by different people; MS analyzed TPE Ghana-051, while LM analyzed the TPE CDC 2575 genome. Both sequencing projects (i.e., TPE Ghana-051 and TPE CDC 2575) revealed identical consensus genome sequences, except for regions showing intrastrain heterogeneity, including *tpr*K variable regions. The basic characteristics of the TPE Ghana-051 and TPE CDC 2575 genomes are shown in Table 1, and these are identical or highly similar to other completely sequenced TPE genomes [2–3]. The TPE Ghana-051 and CDC 2575 strains clustered with TPE strains isolated from Africa, especially with the TPE Gauthier strain (Fig 2A), which differed from it in 129 nucleotide positions (S3 Table).

**Table 1:** Basic characteristics of the TPE Ghana-051 and TPE CDC 2575 genomes and their comparison to published TPE CDC-2 and Gauthier genomes.

Genome parameter	CDC 2575/Ghana-051	CDC-2	Gauthier
GenBank Accession No.	CP020366/CP020365	CP002375.1	CP002376.1
Genome size	1,139,577 bp	1,139,744 bp	1,139,417 bp
G+C content	52.77%	52.80%	52.80%
No. of predicted genes	1124 including 54 untranslated genes	1125 including 54 untranslated genes	1125 including 54 untranslated genes
Sum of the intergenic region lengths (% of the genome length)	53,628 bp (4.71%)	52,963 bp (4.65%)	53,300 bp (4.68%)
Average/median gene length	980.0/830.0 bp	980.4/831.0 bp	979.3/831.0 bp
Average/median gene length of genes with unknown function	818.1/651.5 bp	843.8/657 bp	841.4/652.5 bp
No. of genes encoded on plus/minus DNA strand	599/525	600/525	600/525
No. of annotated pseudogenes	7	6	6
No. of tRNA loci	45	45	45
No. of rRNA loci	6 (2 operons)	6 (2 operons)	6 (2 operons)
No. of ncRNAs	3	3	3

### Intrastrain heterogeneity in TPE Ghana-051 and TPE CDC 2575 genomes

Both TPE Ghana-051 and TPE CDC 2575 genomes were assessed for the presence of intrastrain heterogeneity, as described previously [21]. The frequency of minor alleles was set to at least 8% and the list of heterogeneous sites is shown in Table 2. While the genome of TPE Ghana-051 had 13 heterogeneous sites, the genome of TPE CDC 2575 had 5 heterogeneous sites. Four heterogeneous sites at positions 134948, 522943, 700634, and 997894, relative to strain Samoa D, were found at the same positions in both genomes, but differing in the frequency of both alleles (Table 2). All minor alleles were supported by at least 19 independent Illumina reads with a mean value of the number of minor allele Illumina reads of 278.5. The average sequencing error rates for these positions, calculated from alternative reads (i.e., other than minor and major alleles) for nucleotide positions (presented in Table 2) was 0.094%. Therefore, a cutoff of 8% for alternative reads was more than two orders of magnitude higher than the average error rate of Illumina reads for these positions.

**Table 2:** Intrastrain heterogeneity found in TPE Ghana-051 and TPE CDC 2575 genomes. Minor alleles with a frequency over 8% are shown.

Samoa D (CP002374.1) coordinates	TPE CDC 2575					TPE Ghana-051							
	SNV*	**Coverage**	**A**	**C**	**G**		**T**	**SNV**	**Coverage**	**A**	**C**	**G**	**T**
			**N**^**§**^ **(%)**	**N (%)**	**N (%)**		**N (%)**			**N (%)**	**N (%)**	**N (%)**	**N (%)**
**134948**	**T/C**	2855	1	634	1		2219	**C/T**	2336	0	1234	0	1102
							**(77.7)**				**(52.8)**		
**165819**	**T/A**	147	19	0	0		128	**T**	822	0	0	0	822
							**(87.1)**						**(100.0)**
**218783**	**G**	1788	0	0	1788		0	**G/T**	1259	1	0	1068	190
					**(100.0)**							**(84.8)**	
**235835**	**A**	1830	1830	0	0		0	**A/G**	3003	2105	1	897	0
			**(100.0)**							**(70.1)**			
**522943**	**C/T**	328	0	258	0		70	**C/T**	581	0	423	0	158
				**(78.7)**							**(72.8)**		
**623679**	**A**	1007	1007	0	0		0	**A/G**	1094	983	0	110	1
			**(100.0)**							**(89.9)**			
**653791**	**A**	1905	1905	0	0		0	**A/G**	13333	10672	3	2642	16
			**(100.0)**							**(80.0)**			
**700634**	**C/T**	1360	0	1058	0		302	**T/C**	3541	1	1618	1	1921
				**(77.8)**									**(54.3)**
**821808**	**A**	3070	3070	0	0		0	**A/G**	2397	2158	0	236	3
			**(100.0)**							**(90.0)**			
**843235**	**G**	165	0	0	165		0	**G/C**	931	0	167	764	0
					**(100.0)**							**(82.1)**	
**987804**	**C**	2023	0	2023	0		0	**C/T**	1829	1	1646	0	182
				**(100.0)**							**(90.0)**		
**997894**	**G/A**	5953	2501	1	3449		2	**A/G**	6334	5010	0	1322	2
					**(57.9)**					**(79.1)**			
**1051146**	**T**	2185	0	0	0		2185	**T/C**	2812	2	415	0	2395
							**(100.0)**						**(85.2)**
**1093898**	**C**	2161	0	2161	0		0	**C/A**	1251	255	996	0	0
				**(100.0)**							**(79.6)**		

*SNV, single nucleotide variant (major/minor allele); N^§^ = number of individual reads

One of the 14 polymorphic sites (235835) was in an intergenic region. Most of the alternative alleles (11 out of 13) resulted in non-synonymous mutations in genes encoding proteins involved in substrate transport and metabolism (Table 3).

**Table 3:** Treponemal genes with detected intrastrain heterogeneity.

Gene #	Gene name	Gene coordinate	Samoa D (CP002374.1) coordinate	Protein function	CDC 2575	Ghana-051	Type of amino acid change
TP0117	*tprC*	134598–136394	134948	TprC	T/C	C/T	non-synonymous
TP0144	*tbpA*	165548–166555	165819	thiamine ABC superfamily ATP binding cassette transporter, binding protein	T/A	T	non-synonymous
TP0215	*grpE*	218732–219394	218783	chaperone GrpE	G	G/T	non-synonymous
IGR*	NA**	NA	235835	IGR *rrf1* (5S ribosomal RNA)—TP0226 (putative membrane protein)	A	A/G	NA
TP0488	*mcp-2*	522387–524924	522943	methyl-accepting chemotaxis protein	C/T	C/T	non-synonymous
TP0572	* *	622652–623734	623679	FMN-binding domain protein, putative membrane protein	A	A/G	non-synonymous
TP0600	* *	653165–654517	653791	putative membrane-associated zinc protease	A	A/G	non-synonymous
TP0639	*mcp-3*	699340–701274	700634	methyl-accepting chemotaxis protein	C/T	T/C	non-synonymous
TP0755	*ptsN*	821173–821829	821808	PTS family fructose/mannitol (*fru*) porter component IIA	A	A/G	non-synonymous
TP0773	*htrA1*	842183–843415	843235	S1 family peptidase Do	G	G/C	non-synonymous
TP0903	*murD*	986570–988168	987804	UDP-N-acetylmuramoyl-L-alanine—D-glutamate ligase	C	C/T	non-synonymous
TP0919		997862–998179	997894	thioredoxin	G/A	A/G	non-synonymous
TP0967		1050909–1052456	1051146	hypothetical protein	T	T/C	synonymous
TP1003		1093188–1094225	1093898	hypothetical protein, putative membrane protein	C	C/A	synonymous

*IGR, intergenic region

**NA, not applicable

### Molecular analysis of rabbit chromosomal DNA in Ghana-051 and CDC 2575 samples

Since consensus genome sequences of both TPE samples were identical (although both strains contained several intrastrain heterogeneous sites) and since these samples were also contaminated by rabbit DNA derived from the experimentally inoculated rabbits, the rabbit chromosomal DNA in Ghana-051 and CDC 2575 samples was analyzed to verify the independent character of both samples. Altogether, sequencing of Ghana-051 and CDC 2575 resulted in 2,150,741 and 1,731,654 reads aligning to Chromosome 1 rabbit DNA, respectively. Altogether, 21 positions in rabbit DNA were different between Ghana-051 and CDC 2575 samples. From these reads, a region differing in 7 nucleotide positions and spanning less than 1 kb was selected, amplified, and analyzed in additional TPE samples from the same laboratory and time period. The sequenced region covered positions 53848036–53849014 in the genome of *Oryctolagus cuniculus* (CM000790.1) (Table 4), i.e., the region between the DMRT1 gene (encoding Doublesex and mab-3 related transcription factor 1; coordinates 53687806–53804010) and the KANK1 gene (encoding the KN motif and ankyrin repeat domain-containing protein 1; coordinates 53898320–53958377). Within this region, 46 and 141 Illumina sequencing reads were mapped to the rabbit reference in the Ghana-051 and CDC 2575 samples, respectively. Another TPE sample that was propagated in rabbits in the same laboratory and at about the same time (1990; TPE Sei Geringging, [22]) showed sequences in these nucleotide positions that were identical with the rabbit DNA from the TPE Ghana-051 sample (1988). Similarly, the TPE CDC-2 sample (isolated in 1980, [8]) showed identical rabbit sequences to the rabbit sequences from the CDC 2575 sample (isolated in 1980).

**Table 4:** Differences between rabbit chromosomal DNA of New Zealand White rabbits in the Ghana-051, CDC 2575, Sei Geringging, and CDC-2 samples.

Rabbit samples containing *Treponema pallidum* subsp. *pertenue*				Differences in rabbit chromosomal DNA*						
**TPE Sample**	Place of isolation	Year of isolation	Reference	53848160**	53848292**	53848328**	53848372**	53848487**	53848489**	53848574**
**CDC-2**	Ghana	1980	[8]	A	T	G	T	A	A	G
**CDC 2575**	Ghana	1980	[8, 9]	A	T	G	T	A	A	G
**Ghana-051**	Ghana	1988	[10]	G	C	A	C	C	C	A
**Sei Geringging K403**	Indonesia	1990	[22]	G	C	A	C	C	C	A

*Differences in the DNA sequence of chromosome 1 of *Oryctolagus cuniculus* based on reads/amplicons from the original sample

**Position in the genome of *Oryctolagus cuniculus* according to the reference sequence CM000790.1

### Estimation of the upper limit of the mutation rate in yaws treponemes

There was a time span of 7 years and 3 months between isolation of the two strains and, during this time, the TPE strain Ghana-051 multiplied in infected humans. Using this time period (7.25 years) and the number of detected mutations (n = 0), the upper limit of the mutation rate in yaws treponemes was estimated. The mutation rate (μ) per site per year can be calculated using the following formula:

$$\mu =\frac{n}{t\;x\;gl}$$

where n means the number of nucleotide differences, t means the time since the samples have diverged measured in years, and gl means the number of nucleotide positions (genome length). For the number of nucleotide positions, a genome size of 1,139,577 nt was used. Since sites with intrastrain heterogeneity do not represent fixed mutations, they were not used for the estimation of the TPE mutation rate. No mutations during 7.25 years corresponds to a mutation rate of 1.21 x 10^−7^ per nucleotide site per year or lower. Estimation of a mutation rate (μ) per site per generation was calculated from this formula:

$$\mu =\frac{n}{ng\;x\;gl}$$

where ng means the number of generations during the time the samples diverged. Considering the doubling time of yaws spirochetes, about 30 hours [23, 24], and the optimal proliferating conditions during the entire 7.25 year period, one can assume that 63,510 hours (7.25 years) between the isolation of the two samples corresponded to 2,117 treponemal generations. This number corresponds to a TPE mutation rate of 4.1 x 10^−10^ per site per generation (assuming 292 generations per year).

## Discussion

Two TPE strains isolated more than 7 years apart from each other, from young patients who were originally infected in Ghana, Africa, were analyzed and their genomes were completely sequenced with PSGS [2–4, 11]. The consensus genome sequences were identical despite the fact that the isolation of DNA, genome amplifications, sequence assembly, and genome analyses were performed separately at different times and by different people. This finding indicates that (1) there is a very low error rate in PSGS sequencing, i.e., on the order of 10^−6^ or lower and (2) the genomes of uncultivable pathogenic treponemes are quite stable over time.

The identical consensus whole genome sequences raised the question as to whether the Ghana-051 and CDC 2575 samples really represented two different strains. There are several lines of evidence that indicate that the TPE Ghana-051 and TPE CDC 2575 samples are different and do not represent a single, duplicated sample. First, treponemes sequenced from the two samples showed differences in 14 intrastrain heterogeneous sites (either in the positions of the heterogeneous sites or in the frequency of the detected nucleotide variants), indicating that the population of treponemes present in the two samples were different. At the same time, 4 common positions of intrastrain heterogeneous sites suggested that treponemes from both samples were highly similar and originated from a common ancestor population. Second, the contaminating chromosomal rabbit DNA in both samples (between the DMRT1 and KANK1 genes) differed, and the observed nucleotide sequences in Ghana-051 were similar to samples from the same time period and the same laboratory, i.e., to TPE Sei Geringging, which was also propagated in the Netherlands. At the same time, the rabbit DNA in CDC 2575 was identical to the tested nucleotide positions in the CDC-2 sample, which was isolated in Ghana at the same time. These data clearly indicate that both Ghana-051 and CDC 2575 represented different samples with rabbit DNA sequences related to samples from a similar time period and place of isolation. Third, TPE CDC 2575 was provided to Dr. Noordhoek by Dr. Peter Perine from CDC and this strain was used in the study by Noordhoek et al. [9], even before isolation of TPE Ghana-051. A potential mislabeling or swapping of TPE CDC 2575 with other two TPE strains, CDC-1 and CDC-2, isolated in Ghana in 1980 and described in Liska et al. [8] can be excluded since genome sequence of CDC 2575 differs from CDC-2 [2] as well as from CDC-1 according to an ongoing sequencing project in our laboratory. Fourth, TPE Ghana-051 was provided to Dr. Noordhoek from Sophia Children's Hospital in Rotterdam, The Netherlands, and according to the Dr. Noordhoek's lab record, this sample was described in the study by Engelkens et al. [10]. The sequenced sample of TPE Ghana-051 showed similar signatures of rabbit DNA as rabbits used in the Netherlands at the same time period (1988) and the original tube from which the TPE Ghana-051 was sequenced originated from 1989. Both these facts support strain TPE Ghana-051 as the original sample isolated in the Netherlands. Taken together, the possible duplication of either CDC 2575 or Ghana-051 samples can be excluded.

Several previous studies [25–27] described partial sequences of CDC 2575 (n = 24) and Ghana-051 (n = 22) comprising 8,733 bp and 7,583 bp, respectively. Altogether, 5 nucleotide discrepancies, located in genes TP0617 (1 nucleotide difference; GenBank Acc. No. EU101912.1 for CDC-2575 and EU101917.1 for Ghana-051) and TP0620 (4 nucleotide differences; GenBank Acc. No. JN582339.1 for CDC 2575), were identified. However, the TPE CDC 2575 sequence of TP0620 (JN582339.1), published in a study by Pillay et al. [27], was likely mislabeled since this sequence was also identical to the TP0620 of the TPA Nichols strain, which had also been used in the aforementioned study [27]. The remaining difference (C vs. T) at position 671367 corresponding to the complete genome sequence of CDC 2575 and Ghana-051 in the TP0617 gene was analyzed in individual sequencing reads and revealed a cytosine at this position, which was supported by 97.9% (1266x coverage) and 96.9% (2080x coverage) of the reads in CDC 2575 and Ghana-051, respectively. A thymine at this position was detected in 2.1% and 2.9% of the reads in CDC 2575 and Ghana-051, respectively, suggesting that the observed difference may represent an intrastrain heterogeneous site, a sequencing error, or both.

Genetic diversity within individual treponemal strains, i.e. intrastrain genetic diversity, was detected in several previous studies and mostly affected *tpr* genes and sequences in their vicinity or sequences paralogous to *tpr* genes [28–34]. A previous genome-wide study [21] on genetic heterogeneity revealed 17 genes in 5 treponemal genomes with 23 intrastrain heterogeneous sites; the number of identified heterogeneous sites in individual genomes varied between 0 and 7. However, the average sequence coverage of analyzed genomes in the previous study [21] ranged between 21x and 194x, while in the present study the coverage was well over 800x, which may explain the higher detection rate of intrastrain variable sites. Another recent genome-wide study [35] revealed 63 intrastrain heterogeneous sites in 25 TPA genomes. The median average depth of coverage of the genomes analyzed in this study [35] was also relatively high compared to the original study [21] and reached 131x (ranging from 20x to 1,196x). While it is clear that the number of identified intrastrain heterogeneous sites somehow correlates with the average depth of coverage, it is not clear if the number of heterogeneous sites also reflects the *T*. *pallidum* subspecies, as suggested by Čejková et al. [21], where the majority of heterogeneous sites were found among TPA strains and not among TPE strains. As with the previously identified heterogeneous sites, the alternative alleles primarily encoded non-synonymous replacements of amino acid residues in the corresponding protein sequences, suggesting an adaptive character for this genetic variability. Interestingly, TPE strain Ghana-051 showed more heterogeneous sites (n = 13) compared to TPE strain CDC 2575 (n = 5). A previous study showed that when DNA preparations originated from two different rabbit passages, the relative proportions of alleles differed [21]. It is therefore not clear if the observed differences in occurrence and frequency of heterogeneous sites reflect differences associated with when (i.e., the year) the patient’s sample was isolated, or if it was the differences in the number of hamster and rabbit passages, or both.

Seven years and 3 months elapsed between the isolation of the two samples and this corresponds to a mutation rate of 1.21 x 10^−7^ per TPE nucleotide site per year or lower or 4.1 x 10^−10^ per site per generation (assuming 292 generations per year). For our calculations, we omitted the time period over which the two TPE strains evolved separately, i.e., the time between TPE infection and the appearance of clinical symptoms in the patient that originated the older sample (CDC 2575), and also the time that was needed for isolation of both strains during experimental hamster and rabbit infection. The reason for this was the lack of data indicating the exact time of infection and the exact number and duration of animal passages. However, both these factors would act to further lower the calculated mutation rate. In *E*. *coli*, an *in vitro* experiment revealed about 100 fixed genome mutations per 40,000 generations, i.e., one mutation per approximately 400 generations [36]. As shown by Maughan [37], differences in generation time between *E*. *coli* and TPE do not have to affect the evolutionary rates of other organisms with different generation times. The genome size of *E*. *coli* is 4,629,812 nt, i.e., 4.06 times larger than the TPE genomes analyzed in this study and the corresponding mutation rate corresponds to 5.3 x 10^−10^ per site per generation. Interestingly, the mutation rates calculated for both organisms were surprisingly similar. However, in TPE, this estimate represents the upper limit of the mutation rate, which is likely to be lower. Based on paleo-pathological findings and phylogenetic analyses, de Melo et al. [6] estimated the evolutionary rate of syphilis treponemes to be 8.82 x 10^−8^ substitutions per site per year, i.e., a number corresponding to 3.3 x 10^−10^ per site per generation. This estimate is close to the highest substitution rate revealed by the present study. Moreover, the mutation rates in microbes with DNA-based chromosomes were estimated to be close to 1/300 per genome per replication [38], i.e., 2.92 x 10^−9^ per site per generation, less than an order of magnitude higher than that estimated in this study. Altogether, the estimated upper limit of the TPE mutation rate appears to be close to that of other bacteria and to other mutation rate predictions in treponemes (based on different data). Since the evolutionary rate differs between bacterial species [39], it is not clear how the estimated upper limit of the TPE mutation rate is applicable to related treponemal subspecies, including syphilis treponemes (TPA), TEN, and *T*. *paraluisleporidarum* [40] treponemes. Uncultivable pathogenic treponemes are monomorphic bacteria [5] that have extremely high sequence similarity; therefore, it is likely that other related treponemes show similar mutation rates. However, a study published by Arora et al. [7] estimated the mean evolutionary rate of TPA as 6.6 x 10^−7^ substitutions per site per year, which was based on sample isolation dates and a Birth-death serial skyline model [41]. This value is about 5.5 times higher than the upper limit estimated in this study (1.21 x 10^−7^ per nucleotide site per year). The differences in the estimated TPA error rate could be related to the model used and/or the differences between TPA and TPE mutation rates.

Several studies showed that non-human primates can represent possible reservoirs of human yaws [42–46]. Since yaws treponemes appear to be genetically stable for years, molecular typing of yaws-causing strains will help in future to prove or exclude a transfer of yaws strains between non-human primates and humans. Moreover, epidemiological mapping of TPE strains could help to identify transmission networks also within humans which might be valuable especially in recent yaws eradication efforts. In 2012, the WHO launched a plan for eradication of yaws using macrolide antibiotic azithromycin [47]. So far, azithromycin was found to be effective in treatment of yaws [48–51], although in syphilis-causing strains, two mutation were associated with macrolide resistance [52–53]. Since resistance to azithromycin is caused by point mutation in the 23S rRNA gene and since both copies of the 23S rRNA genes need to be mutated, we have previously estimated the probability of emergence of macrolide-resistant yaws treponemes to be a rather rare event (with probability 10^−4^–10^−5^ per infected patient) [54].

Another implication of the yaws treponemes genome stability during human infections is the possible implication for syphilis treponemes typing studies [54–60], where the stability of *arp*, *tprEGJ*, TP0136, TP0548, and 23S rRNA loci were repeatedly discussed. If the major finding of this study could be extrapolated to TPA strains, then all loci used, so far, for molecular typing of syphilis treponemes, should be sufficiently stable to infer epidemiological relationships.

There are over 2,000 nucleotide differences between TPE and TPA strains [2] and the majority of them are scattered throughout the genomes with only a minority of loci positively selected or recombinant. Based on the known genetic diversity between TPA and TPE genomes, the observation-derived estimate of the upper limit of the mutation rate, and the assumption that the mutation rate in syphilis treponemes is similar to that of TPE, the most recent common ancestor of the syphilis and yaws treponemes is more than ten thousand years old. Assuming an example that uses a 10-times lower mutation rate compared to the upper limit revealed in this study, the most recent common ancestor of syphilis and yaws treponemes would have appeared at a time that would have fallen within the beginning of evolution of modern humans. Taken together, the data presented in this study are consistent with the relatively slow evolution rate of yaws treponemes and suggest that the appearance of the most recent common ancestor of syphilis and yaws treponemes took a very long time, perhaps even a length of time comparable to the evolution of modern humans.

## Supporting information

S1 Table List of primers used for amplification of TP intervals of CDC 2575 and Ghana-051 strains using the pool segment genome sequencing (PSGS) approach. (XLS)

S2 Table Genome coverage statistics for TPE CDC 2575 and Ghana-051 strains. Illumina sequencing reads were mapped to the Samoa D reference genome (CP002374.1).
(XLS)

S3 Table SNV analysis for TPE Ghana-051 and CDC 2575 strains. S3A. SNV analysis for Gauthier and Ghana-051 / CDC 2575 genomes. S3B: SNV analysis for CDC-2 and Ghana-051 / CDC 2575 genomes.
(XLS)

## References

[1] Giacani L, Lukehart SA. The endemic treponematoses. Clin Microbiol Rev. 2014;27(1): 89–115. doi: 10.1128/CMR.00070-13 24396138 10.1128/CMR.00070-13 PMC3910905

[2] Čejková D, Zobaníková M, Chen L, Pospíšilová P, Strouhal M, Qin X, et al Whole genome sequences of three *Treponema pallidum* ssp. *pertenue* strains: yaws and syphilis treponemes differ in less than 0.2% of the genome sequence. PLoS Negl Trop Dis. 2012;6(1): e1471 doi: 10.1371/journal.pntd.0001471 22292095 10.1371/journal.pntd.0001471 PMC3265458

[3] Zobaníková M, Strouhal M, Mikalová L, Čejková D, Ambrožová L, Pospíšilová P, et al Whole genome sequence of the *Treponema* Fribourg-Blanc: unspecified simian isolate is highly similar to the yaws subspecies. PLoS Negl Trop Dis. 2013;7(4): e2172 doi: 10.1371/journal.pntd.0002172 23638193 10.1371/journal.pntd.0002172 PMC3630124

[4] Štaudová B, Strouhal M, Zobaníková M, Čejková D, Fulton LL, Chen L, et al Whole genome sequence of the *Treponema pallidum* subsp. *endemicum* strain Bosnia A: the genome is related to yaws treponemes but contains few loci similar to syphilis treponemes. PLoS Negl Trop Dis. 2014;8(11): e3261 doi: 10.1371/journal.pntd.0003261 10.1371/journal.pntd.0003261 PMC4222731 25375929

[5] Šmajs D, Norris SJ, Weinstock GM. Genetic diversity in *Treponema pallidum*: implications for pathogenesis, evolution and molecular diagnostics of syphilis and yaws. Infect Genet Evol. 2012;12(2): 191–202. doi: 10.1016/j.meegid.2011.12.001 22198325 10.1016/j.meegid.2011.12.001 PMC3786143

[6] de Melo FL, de Mello JC, Fraga AM, Nunes K, Eggers S. Syphilis at the crossroad of phylogenetics and paleopathology. PLoS Negl Trop Dis. 2010;4(1): e575 doi: 10.1371/journal.pntd.0000575 20052268 10.1371/journal.pntd.0000575 PMC2793018

[7] Arora N, Schuenemann VJ, Jäger G, Peltzer A, Seitz A, Herbig A, et al Origin of modern syphilis and emergence of a pandemic *Treponema pallidum* cluster. Nat Microbiol. 2016;2: 16245 doi: 10.1038/nmicrobiol.2016.245 27918528 10.1038/nmicrobiol.2016.245

[8] Liska SL, Perine PL, Hunter EF, Crawford JA, Feeley JC. Isolation and transportation of *Treponema pertenue* in golden hamsters. Curr Microbiol. 1982;7(1): 41–43.

[9] Noordhoek GT, Hermans PW, Paul AN, Schouls LM, van der Sluis JJ, van Embden JD. *Treponema pallidum* subspecies *pallidum* (Nichols) and *Treponema pallidum* subspecies *pertenue* (CDC 2575) differ in at least one nucleotide: comparison of two homologous antigens. Microb Pathog. 1989;6(1): 29–42. 2471912 10.1016/0882-4010(89)90005-3

[10] Engelkens HJ, Oranje AP, Stolz E. Early yaws, imported in The Netherlands. Genitourin Med. 1989;65(5): 316–318. 2583714 10.1136/sti.65.5.316 PMC1194384

[11] Weinstock GM, Šmajs D, Hardham J, Norris SJ. From microbial genome sequence to applications. Res Microbiol. 2000;151(2): 151–158. 10865961 10.1016/s0923-2508(00)00115-7

[12] Bolger AM, Lohse M, Usadel B. Trimmomatic: a flexible trimmer for Illumina sequence data. Bioinformatics. 2014;30(15): 2114–2120. doi: 10.1093/bioinformatics/btu170 24695404 10.1093/bioinformatics/btu170 PMC4103590

[13] Kearse M, Moir R, Wilson A, Stones-Havas S, Cheung M, Sturrock S, et al Geneious Basic: an integrated and extendable desktop software platform for the organization and analysis of sequence data. Bioinformatics. 2012;28(12): 1647–1649. doi: 10.1093/bioinformatics/bts199 22543367 10.1093/bioinformatics/bts199 PMC3371832

[14] Heymans R, Kolader ME, van der Helm JJ, Coutinho RA, Bruisten SM. *TprK* gene regions are not suitable for epidemiological syphilis typing. Eur J Clin Microbiol Infect Dis. 2009;28(7): 875–878. doi: 10.1007/s10096-009-0717-5 19229562 10.1007/s10096-009-0717-5

[15] Kumar S, Stecher G, Tamura K. MEGA7: molecular evolutionary genetics analysis version 7.0 for bigger datasets. Mol Biol Evol. 2016;33: 1870–1874. doi: 10.1093/molbev/msw054 27004904 10.1093/molbev/msw054 PMC8210823

[16] Li H. Aligning sequence reads, clone sequences and assembly contigs with BWA-MEM. ArXiv e-Prints 2013;1303:3997.

[17] Li H, Handsaker B, Wysoker A, Fennell T, Ruan J, Homer N, et al The sequence alignment/map format and SAMtools. Bioinformatics. 2009;25(16): 2078–2079. doi: 10.1093/bioinformatics/btp352 19505943 10.1093/bioinformatics/btp352 PMC2723002

[18] Koboldt DC, Chen K, Wylie T, Larson DE, McLellan MD, Mardis ER, et al VarScan: variant detection in massively parallel sequencing of individual and pooled samples. Bioinformatics. 2009;25(17): 2283–2285. doi: 10.1093/bioinformatics/btp373 19542151 10.1093/bioinformatics/btp373 PMC2734323

[19] Čejková D., Zobaníková M., Pospíšilová P., Strouhal M., Mikalová L., Weinstock G.M., et al Structure of *rrn* operons in pathogenic non-cultivable treponemes: sequence but not genomic position of intergenic spacers correlates with classification of *Treponema pallidum* and *T*. *paraluiscuniculi* strains. J Med Microbiol. 2013;62(2): 196–207. 23082031 10.1099/jmm.0.050658-0 PMC3755535

[20] Centurion-Lara A, Giacani L, Godornes C, Molini BJ, Brinck Reid T, Lukehart SA. Fine analysis of genetic diversity of the *tpr* gene family among treponemal species, subspecies and strains. PLoS Negl Trop Dis. 2013; 7(5):e2222 doi: 10.1371/journal.pntd.0002222 23696912 10.1371/journal.pntd.0002222 PMC3656149

[21] Čejková D, Strouhal M, Norris SJ, Weinstock GM, Šmajs D. A Retrospective study on genetic heterogeneity within *Treponema* strains: subpopulations are genetically distinct in a limited number of positions. PLoS Negl Trop Dis. 2015;9(10): e0004110 doi: 10.1371/journal.pntd.0004110 26436423 10.1371/journal.pntd.0004110 PMC4593590

[22] Noordhoek GT, Engelkens HJ, Judanarso J, van der Stek J, Aelbers GN, van der Sluis JJ, et al Yaws in West Sumatra, Indonesia: clinical manifestations, serological findings and characterization of new *Treponema* isolates by DNA probes. Eur J Clin Microbiol Infect Dis. 1991;10(1): 12–19. 2009873 10.1007/BF01967091

[23] Cumberland MC, Turner TB. Rate of multiplication of *Treponema pallidum* in normal and immune rabbits. Am J Syphilis. 1949;33(3): 201–212. 18121293

[24] Magnuson HJ, Eagle H, Fleischmann R. The minimal infectious inoculum of *Spirochaeta pallida* (Nichols strain), and a consideration of its rate of multiplication in vivo. Am J Syph Gonorrhea Vener Dis. 1948;32(1): 1–18. 18917621

[25] Harper KN, Liu H, Ocampo PS, Steiner BM, Martin A, Levert K, et al The sequence of the acidic repeat protein (*arp*) gene differentiates venereal from nonvenereal *Treponema pallidum* subspecies, and the gene has evolved under strong positive selection in the subspecies that causes syphilis. FEMS Immunol Med Microbiol. 2008;53(3): 322–332. doi: 10.1111/j.1574-695X.2008.00427.x 18554302 10.1111/j.1574-695X.2008.00427.x

[26] Harper KN, Ocampo PS, Steiner BM, George RW, Silverman MS, Bolotin S, et al On the origin of the treponematoses: a phylogenetic approach. PLoS Negl Trop Dis. 2008;2(1): e148 doi: 10.1371/journal.pntd.0000148 18235852 10.1371/journal.pntd.0000148 PMC2217670

[27] Pillay A, Chen CY, Reynolds MG, Mombouli JV, Castro AC, Louvouezo D, et al Laboratory-confirmed case of yaws in a 10-year-old boy from the Republic of the Congo. J Clin Microbiol. 2011;49(11): 4013–4015. doi: 10.1128/JCM.01121-11 21918034 10.1128/JCM.01121-11 PMC3209121

[28] Stamm LV, Bergen HL. The sequence-variable, single-copy *tprK* gene of *Treponema pallidum* Nichols strain UNC and Street strain 14 encodes heterogeneous TprK proteins. Infect Immun. 2000;68(11): 6482–6486. 11035764 10.1128/iai.68.11.6482-6486.2000 PMC97738

[29] Centurion-Lara A, Godornes C, Castro C, Van Voorhis WC, Lukehart SA. The *tprK* gene is heterogeneous among *Treponema pallidum* strains and has multiple alleles. Infect Immun. 2000;68(2): 824–831. 10639452 10.1128/iai.68.2.824-831.2000 PMC97211

[30] Šmajs D, McKevitt M, Wang L, Howell JK, Norris SJ, Palzkill T, et al BAC library of *T*. *pallidum* DNA in *E*. *coli*. Genome Res. 2002;12(3): 515–522. doi: 10.1101/gr.207302 11875041 10.1101/gr.207302 PMC155280

[31] LaFond RE, Centurion-Lara A, Godornes C, Rompalo AM, Van Voorhis WC, Lukehart SA. Sequence diversity of *Treponema pallidum* subsp. *pallidum tprK* in human syphilis lesions and rabbit-propagated isolates. J Bacteriol. 2003;185(21): 6262–6268. doi: 10.1128/JB.185.21.6262-6268.2003 14563860 10.1128/JB.185.21.6262-6268.2003 PMC219401

[32] Matějková P, Strouhal M, Šmajs D, Norris SJ, Palzkill T, Petrosino JF, et al Complete genome sequence of *Treponema pallidum* ssp. *pallidum* strain SS14 determined with oligonucleotide arrays. BMC Microbiol. 2008;8: 76 doi: 10.1186/1471-2180-8-76 18482458 10.1186/1471-2180-8-76 PMC2408589

[33] Giacani L, Brandt SL, Puray-Chavez M, Reid TB, Godornes C, Molini BJ, et al Comparative investigation of the genomic regions involved in antigenic variation of the TprK antigen among treponemal species, subspecies, and strains. J Bacteriol. 2012;194(16): 4208–4225. doi: 10.1128/JB.00863-12 22661689 10.1128/JB.00863-12 PMC3416249

[34] Pětrošová H, Pospíšilová P, Strouhal M, Čejková D, Zobaníková M, Mikalová L, et al Resequencing of *Treponema pallidum* ssp. *pallidum* strains Nichols and SS14: correction of sequencing errors resulted in increased separation of syphilis treponeme subclusters. PLoS One. 2013;8(9): e74319 doi: 10.1371/journal.pone.0074319 24058545 10.1371/journal.pone.0074319 PMC3769245

[35] Pinto M, Borges V, Antelo M, Pinheiro M, Nunes A, Azevedo J, et al Genome-scale analysis of the non-cultivable *Treponema pallidum* reveals extensive within-patient genetic variation. Nat Microbiol. 2016;2: 16190 doi: 10.1038/nmicrobiol.2016.190 27748767 10.1038/nmicrobiol.2016.190

[36] Blount ZD, Barrick JE, Davidson CJ, Lenski RE. Genomic analysis of a key innovation in an experimental *Escherichia coli* population. Nature. 2012;489(7417): 513–518. doi: 10.1038/nature11514 22992527 10.1038/nature11514 PMC3461117

[37] Maughan H. Rates of molecular evolution in bacteria are relatively constant despite spore dormancy. Evolution. 2007;61(2): 280–288. doi: 10.1111/j.1558-5646.2007.00026.x 17348939 10.1111/j.1558-5646.2007.00026.x

[38] Drake JW, Charlesworth B, Charlesworth D, Crow JF. Rates of spontaneous mutation. Genetics. 1998;148(4): 1667–1686. 9560386 10.1093/genetics/148.4.1667 PMC1460098

[39] Ochman H, Elwyn S, Moran NA. Calibrating bacterial evolution. Proc Natl Acad Sci U S A. 1999;96(22): 12638–12643. 10535975 10.1073/pnas.96.22.12638 PMC23026

[40] Lumeij JT, Mikalová L, Šmajs D. Is there a difference between hare syphilis and rabbit syphilis? Cross infection experiments between rabbits and hares. Vet Microbiol. 2013;164(1–2): 190–194. doi: 10.1016/j.vetmic.2013.02.001 23473645 10.1016/j.vetmic.2013.02.001

[41] Stadler T, Kühnert D, Bonhoeffer S, Drummond AJ. Birth-death skyline plot reveals temporal changes of epidemic spread in HIV and hepatitis C virus (HCV). Proc Natl Acad Sci U S A. 2013;110(1): 228–233. doi: 10.1073/pnas.1207965110 23248286 10.1073/pnas.1207965110 PMC3538216

[42] Levréro F, Gatti S, Gautier-Hion A, Ménard N. Yaws disease in a wild gorilla population and its impact on the reproductive status of males. Am J Phys Anthropol. 2007;132(4):568–575. doi: 10.1002/ajpa.20560 17274014 10.1002/ajpa.20560

[43] Knauf S, Batamuzi EK, Mlengeya T, Kilewo M, Lejora IA, Nordhoff M, et al *Treponema* infection associated with genital ulceration in wild baboons. Vet Pathol. 2012;49(2): 292–303. doi: 10.1177/0300985811402839 21411621 10.1177/0300985811402839

[44] Knauf S, Liu H, Harper KN. Treponemal infection in nonhuman primates as possible reservoir for human yaws. Emerg Infect Dis. 2013;19(12): 2058–2060. doi: 10.3201/eid1912.130863 24274094 10.3201/eid1912.130863 PMC3840862

[45] Knauf S, Barnett U, Maciej P, Klapproth M, Ndao I, Frischmann S, et al High prevalence of antibodies against the bacterium *Treponema pallidum* in Senegalese Guinea Baboons (*Papio papio*). PLoS One. 2015;10(11): e0143100 doi: 10.1371/journal.pone.0143100 26588087 10.1371/journal.pone.0143100 PMC4654574

[46] Klegarth AR, Ezeonwu CA, Rompis A, Lee BPY, Aggimarangsee N, Chalise M, et al Survey of treponemal infections in free-ranging and captive macaques, 1999–2012. Emerg Infect Dis. 2017;23(5): 816–819. doi: 10.3201/eid2305.161838 28418297 10.3201/eid2305.161838 PMC5403046

[47] Maurice J. WHO plans new yaws eradication campaign. Lancet. 2012;379(9824): 1377–1378. 22509526 10.1016/s0140-6736(12)60581-9

[48] Mitjà O, Hays R, Ipai A, Penias M, Paru R, Fagaho D, et al Single-dose azithromycin versus benzathine benzylpenicillin for treatment of yaws in children in Papua New Guinea: an open-label, non-inferiority, randomised trial. Lancet. 2012;379(9813): 342–347. doi: 10.1016/S0140-6736(11)61624-3 22240407 10.1016/S0140-6736(11)61624-3

[49] Mitjà O, Houinei W, Moses P, Kapa A, Paru R, Hays R, et al Mass treatment with single-dose azithromycin for yaws. N Engl J Med. 2015;372(8): 703–710. doi: 10.1056/NEJMoa1408586 25693010 10.1056/NEJMoa1408586

[50] Ghinai R, El-Duah P, Chi KH, Pillay A, Solomon AW, Bailey RL, et al A cross-sectional study of 'yaws' in districts of Ghana which have previously undertaken azithromycin mass drug administration for trachoma control. PLoS Negl Trop Dis. 2015;9(1): e0003496 doi: 10.1371/journal.pntd.0003496 25632942 10.1371/journal.pntd.0003496 PMC4310597

[51] Kwakye-Maclean C, Agana N, Gyapong J, Nortey P, Adu-Sarkodie Y, Aryee E, et al A single dose oral azithromycin versus intramuscular benzathine penicillin for the treatment of yaws-a randomized non inferiority trial in Ghana. PLoS Negl Trop Dis. 2017;11(1): e0005154 doi: 10.1371/journal.pntd.0005154 28072863 10.1371/journal.pntd.0005154 PMC5224786

[52] Stamm LV, Bergen HL. A point mutation associated with bacterial macrolide resistance is present in both 23S rRNA genes of an erythromycin-resistant *Treponema pallidum* clinical isolate. Antimicrob Agents Chemother. 2000;44(3): 806–807. 10755994 10.1128/aac.44.3.806-807.2000 PMC89774

[53] Matějková P, Flasarová M, Zákoucká H, Bořek M, Křemenová S, Arenberger P, et al Macrolide treatment failure in a case of secondary syphilis: a novel A2059G mutation in the 23S rRNA gene of *Treponema pallidum* subsp. *pallidum*. J Med Microbiol. 2009;58(Pt 6): 832–836. doi: 10.1099/jmm.0.007542-0 19429763 10.1099/jmm.0.007542-0

[54] Šmajs D, Paštěková L, Grillová L. Macrolide resistance in the syphilis spirochete, *Treponema pallidum* ssp. *pallidum*: Can we also expect macrolide-resistant yaws strains? Am J Trop Med Hyg. 2015;93(4): 678–683. doi: 10.4269/ajtmh.15-0316 26217043 10.4269/ajtmh.15-0316 PMC4596581

[55] Pillay A, Liu H, Chen CY, Holloway B, Sturm AW, Steiner B, et al Molecular subtyping of *Treponema pallidum* subspecies pallidum. Sex Transm Dis. 1998;25(8): 408–414. 9773432 10.1097/00007435-199809000-00004

[56] Marra CM, Sahi SK, Tantalo LC, Godornes C, Reid T, Behets F, et al Enhanced molecular typing of *Treponema pallidum*: geographical distribution of strain types and association with neurosyphilis. J Infect Dis. 2010;202(9): 1380–1388. doi: 10.1086/656533 20868271 10.1086/656533 PMC3114648

[57] Flasarová M, Šmajs D, Matějková P, Woznicová V, Heroldová-Dvořáková M, Votava M. Molecular detection and subtyping of *Treponema pallidum* subsp. *pallidum* in clinical specimens. Epidemiol Mikrobiol Imunol. 2006;55(3): 105–111. 16970074

[58] Flasarová M, Pospíšilová P, Mikalová L, Vališová Z, Dastychová E, Strnadel R, et al Sequencing-based molecular typing of *Treponema pallidum* strains in the Czech Republic: all identified genotypes are related to the sequence of the SS14 strain. Acta Derm Venereol. 2012;92(6): 669–674. doi: 10.2340/00015555-1335 22434073 10.2340/00015555-1335

[59] Mikalová L, Pospíšilová P, Woznicová V, Kuklová I, Zákoucká H, Šmajs D. Comparison of CDC and sequence-based molecular typing of syphilis treponemes: *tpr* and *arp* loci are variable in multiple samples from the same patient. BMC Microbiol. 2013;13: 178 doi: 10.1186/1471-2180-13-178 23898829 10.1186/1471-2180-13-178 PMC3735398

[60] Grillová L, Pětrošová H, Mikalová L, Strnadel R, Dastychová E, Kuklová I, et al Molecular typing of *Treponema pallidum* in the Czech Republic during 2011 to 2013: increased prevalence of identified genotypes and of isolates with macrolide resistance. J Clin Microbiol. 2014;52(10): 3693–3700. doi: 10.1128/JCM.01292-14 25100820 10.1128/JCM.01292-14 PMC4187743

